# Job Demands and Resources, Positive and Negative Affect, and Psychological Distress of Social Workers in China

**DOI:** 10.3389/fpsyt.2021.752382

**Published:** 2022-01-18

**Authors:** Chienchung Huang, Xiaoxia Xie, Shannon P. Cheung, Yuqing Zhou

**Affiliations:** ^1^School of Social Work, Rutgers, The State University of New Jersey, New Brunswick, NJ, United States; ^2^Research Institute of Social Development, Southwestern University of Finance and Economics, Chengdu, China

**Keywords:** job demands, resources, positive affect, negative affect, psychological distress, social workers, China

## Abstract

Globally, human service professionals, like social workers, experience significant job demands (JD) which can lead to outcomes like psychological distress, burnout, and high turnover rates. This is especially true in China, where the social work profession has grown substantially in recent decades. Because social workers play a crucial role in supporting vulnerable communities, there is a need to understand how their work conditions affect outcomes like psychological distress. This study applies the job demands and resources (JD-R) model to study this relation, along with the mediational effects of positive affect (PA) and negative affect (NA), in social workers from Chengdu, China (*n* = 897). The results of structural equation modeling indicate that JD-R differentially affect psychological distress. PA and NA partially mediate these relations. Job resources (JR) reduced psychological distress by reducing NA and increasing PA. JD did not have any effect on PA but significantly increased NA, which was associated with higher psychological distress. The magnitudes of each estimate suggest that JR has a greater effect on PA and NA, relative to the effects of JD on PA and NA. Interventions that seek to promote PA and reduce NA may be able to work with existing JR to buffer against the effects of JD in social workers.

## Introduction

Industrialized economies have undergone a significant transformation in workforce structure in past decades, leading to greater job demands and work-related stress (1–4). Work-related stress is associated with a range of health-compromising behaviors which individuals may use to cope with or to manage stress (5–7). It is no surprise, then, that studies have also shown that work-related stress is a significant risk factor of poorer physical (6, 8) and psychological health and well-being (3, 4, 6). While work-related stress is not specific to any singular occupation, research has found that some occupational groups experience greater-than-average work-related stress (7, 9, 10). Social workers, for example, experience a high degree of work-related stress and, therefore, have a greater risk of burnout (7, 10, 11). This stress is often associated with the emotional labor demands required of many who work human service jobs (10, 11). Indeed, compassion fatigue has been found to be a positive predictor of psychological distress among social workers from several cross-cultural studies (12–14). Given this, mediational studies of how job demands like emotional workload or labor—and other work conditions—affect psychological distress in social workers are needed to better understand possible points of intervention and to promote the psychological well-being of social workers, an occupational group which provides critical services to vulnerable populations. The goal of this study is to apply the job demands and resources (JD-R) model to examine how job demands (JD) and job resources (JR) differentially affect psychological distress and whether these relations are parallelly mediated by positive affect (PA) and negative affect (NA) in a sample of Chinese social workers.

## Literature Review

In China, the social work profession has developed rapidly over the last two decades. In <10 years, the workforce increased from 0.2 million in 2010 to 1.2 million in 2018 (15, 16). This unprecedented rate of professionalization was brought upon by increasing social problems that followed the country's economic reform in 1978. While social workers in China provide essential services to vulnerable community members in schools, hospitals, community centers, and other social agencies, the future of the profession is threatened by high burnout and psychological distress rates, comparable to those found in international studies (17, 18).

### The Job Demands and Resources (JD-R) Model

Work-related conditions, such as job demands (JD) and job resources (JR), and their effects on employee outcomes have been studied significantly. Studies often apply the job demands and resources (JD-R) model, which posits a conceptual framework to explain how work conditions affect the work and health outcomes of employees (7, 19, 20). This model divides working conditions into the categories of JD and JR, each with different effects on worker outcomes. JD are those aspects of a job that require sustained physical and/or mental effort from an individual. These aspects result in physiological and psychological costs, such as exhaustion. JD act as stressors that activate a performance-protection strategy which results in “strategy adjustments” such as reduced attention and “fatigue after-effects” (e.g., risky decision making; (21), p. 501). JR, on the other hand, are aspects of the job that can facilitate the achievement of work goals and reduce the physiological and psychological costs of JD (21). Demerouti et al. (21) posited that JD-R affect work and health through two processes. In the first, the demanding aspects of work lead to exhaustion and burnout. In the second, a lack of resources further exacerbates the challenges experienced in meeting JD, leading to withdrawal, disengagement, and burnout.

The JD-R model has been supported by a plethora of studies that examine samples of different occupational groups (19, 20, 22) and various health- and work-related outcome variables, including psychological distress and mental health (7, 23–27). Cross-cultural studies have also applied the JD-R model and found effects of JD and JR on workers, whereby, through an energy depletion process, JD causes fatigue, and, through a motivation process, JR can buffer JD's effects. Importantly, however, studies have also found that a lack of JR may compound the effects of JD (7, 25, 28–31).

High JD can have severe consequences for individuals, considering that JD, stress, and burnout are positively correlated with one another (7, 10, 32). It has been suggested that the emotional labor required of certain high stress jobs, including those in the social work profession, can cause greater occupational stress and, subsequently, several other negative outcomes (10). A study on 55 hospice social workers in the U.S. found that over half (56.4%) of the sample experienced moderate compassion fatigue, while just over one-fifth (21.8%) experienced high compassion fatigue (32). Among social workers across several cultures, compassion fatigue has been found to be a positive predictor of psychological distress (12–14).

### Psychological Distress

Psychological distress is an emotional state that is typically characterized by symptoms of depression and anxiety and even somatic complaints (33). An individual may experience psychological distress when they encounter a stressor that is difficult to cope with or to overcome (33, 34). While stress itself is not inherently negative and can be associated with positive emotions and coping, when an individual fails to cope with stress and experiences psychological distress, they are at risk for a plethora of negative behavioral, health, and work outcomes (35–38). Further, psychological distress is a strong predictor of serious mental illnesses, including mood and anxiety disorders (35, 37), and suicidal behavior (39, 40). It follows, then, that understanding the antecedents of psychological distress among social workers, professionals who work, daily, with vulnerable populations, is of utmost importance to develop appropriate interventions that sustain their well-being and prevent the escalation of psychological distress.

JD and JR have been shown to be related to psychological distress, albeit in opposite directions (18, 24, 28). In a survey of 7,800 people from different occupations, Oshio et al. (24) found JD, such as workload, was positively corelated with psychological distress (*r* = 0.26, *p* < 0.001) and JR, such as coworker support, was negatively correlated with psychological distress (*r* = −0.21, *p* < 0.001). Notably, JD and psychological distress have been found to be positively correlated with one another (*r* = 0.39, *p* < 0.001) in a sample of over 600 social workers in Israel (28), indicating a need to further examine the underlying mechanism between JD-R and psychological distress in social workers.

### Positive Affect and Negative Affect (PA and NA)

Over the years, scholars have come to understand subjective well-being as a multi-dimensional construct. In one conceptualization, subjective well-being is comprised of two independent dimensions (41): positive affect and negative affect (PA and NA). Broadly, affect can be described as the experience of any feeling or emotion (American Psychological Association, 2021). Characteristics related to PA include confidence, optimism, sociability, effective coping, and flexibility (42). Fredrickson's (43) broaden-and-build theory posits that PA may broaden momentary thought-action repertoire, allowing people to accrue enduring personal physical, psychological, and social resources that, in turn, facilitate success. Indeed, PA is associated with success across life domains such as work performance, income, and health, among many others (42). Studies that apply the broaden-and-build theory have indicated that PA significantly reduces emotions and stress symptoms, turnover intentions, maladaptive coping, and depression and anxiety (44, 45).

By contrast, NA is characterized by guilt, anxiety, and fear and is associated with physical and mental health outcomes such as emotion dysregulation and psychiatric symptoms (46–49). Weiss and Cropanzano's (50) affective events theory explains that employees' internal influences (e.g., emotions) and reactions to the work environment can affect job performance and satisfaction. Research has found evidence that is consistent with this theory (49, 51, 52). For example, NA is predictive of workplace deviance, including absenteeism, theft, and poor job performance, as well as low well-being (49, 51, 52). Notably, Chen et al. (49) found that PA and NA mediated the relations between work conditions and well-being. Taken together, these studies, along with others, reflect that PA and NA play a significant role in shaping cognition, behavior, and well-being (53, 54), especially in the workplace. Thus, PA and NA may play a role in the relation between JD-R and psychological distress.

Empirical studies which apply the JD-R model, the broaden-and-build theory, and affective events theory have primarily used Western samples. These studies have shown that JD-R has a significant consequence for psychological distress in individuals. Some evidence suggests that PA and NA may mediate this relation, but the current state of knowledge on PA and NA's mediating effects is still quite preliminary. Further, we currently lack scholarship on whether the relation between JD-R and psychological distress is mediated by PA and NA among human service workers, such as social workers. The present study thus examines the effects of JD-R on psychological distress—and whether PA and NA mediate this relation—in a sample of Chinese social workers. The findings of this paper may contribute to the understanding of how JD-R affect psychological distress through PA and NA in a rapidly developing occupational group with a high turnover rate in China.

## Conceptual Model and Hypotheses

**Figure 1:**
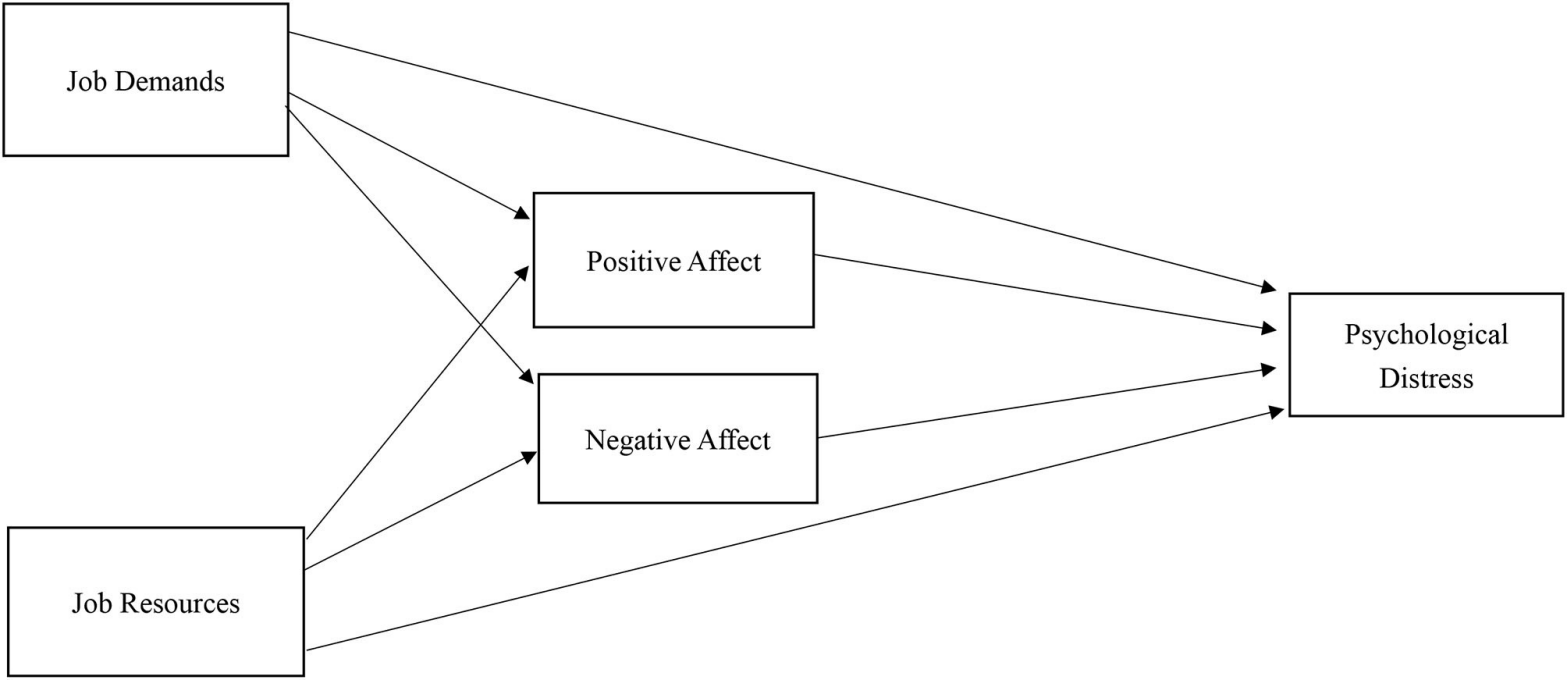
Conceptual model of JD-R, positive affect and negative affect, and psychological distress.

Based on the JD-R framework (21), the broaden-and-build theory (43), and affective events theory (50), a conceptual model involving JD-R, PA, and NA, and psychological distress was proposed to examine the mediational pathways between JD-R and psychological distress *via* PA and NA, as shown in Figure 1. Specifically, we hypothesize that:

1) JD is negatively associated with PA.
2) JD is positively associated with NA.
3) JR is positively associated with PA.
4) JR is negatively associated with NA.
5) PA is negatively associated with psychological distress.
6) NA is positively associated with psychological distress.
7) JD has an indirect effect on psychological distress *via* PA and NA, indicating partial mediation pathways.
8) JR has an indirect effect on psychological distress *via* PA and NA, indicating partial mediation pathways.

## Methods

### Data and Sample

The data for this study were collected from social workers in Chengdu, China, *via* an anonymous web-based survey. Chengdu is the capital city of Sichuan province and has seen rapid development in social work. Within the past decade, the number of employed social workers in Chengdu has multiplied 31.8 times, from 553 in 2010 to 17,622 in 2020 (55). At the same time, however, turnover rates in Chengdu and other big cities have been high, around 20–30% between 2015 and 2020 (56, 57). We randomly selected two districts out of the 22 city districts in Chengdu. We then contacted social work professional associations and agencies that employ social workers within the two districts in order to recruit participants. Each district has around 600 social workers. Members of these organizations were invited to participate in the survey starting May 5, 2021. One and two weeks after the initial invitation, we sent reminders to participate. 915 social workers responded to the survey between May 5, 2021, and May 29, 2021. We excluded 18 surveys from our final analysis due to incomplete data, leaving a final analytic sample of 897. The survey response rate was 75%. An informed consent process was implemented prior to the survey. Participants were compensated 5 RMB (1 USD) after finishing the survey. Further, participants were informed that their participation in the survey was voluntary and that they could choose to discontinue the survey at any point. This research protocol was approved by the institutional review board at one of the co-authors' university in China. About 78% of the sample were female. The average age of the sample was 31.8. A majority of the sample had a at least a college degree (54.6%) and a social work license (52.3%), as shown in Table 1.

**Table 1:** Profile of the sample.

	**Mean (S.D.)**
Gender [%]	
Female	78.3
Male	21.7
Age	31.8 (7.3)
Education achievement [%]	
Below college	45.4
College and above	54.6

*N = 897*.

### Measures

The dependent variable, psychological distress, was assessed by the Kessler 6 Psychological Distress Scale (K6) (37, 58), a 6-item scale that measures psychological distress with high validity and reliability (24, 37, 59). The items in the scale ask respondents about past 30-day prevalence of psychological distress, such as feelings of nervousness, hopelessness, restlessness, worthlessness, and depression. An item in the scale asks about the frequency to which the respondent felt as though “everything was an effort” (37, 58). Items are rated on a 5-point scale which ranges from 0, indicating “none of the time,” to 4, indicating “all of the time.” Psychological distress is represented by the sum of responses to all items, which could range 0 to 24. The severity of psychological distress was according to the K6 has been identified through past calibration studies (35, 58, 60, 61). Scores of 7 and below indicate low psychological distress. Scores between 8 and 12 indicate moderate psychological distress. Scores 13 and above indicate high psychological distress. In this study, the K6 scale had a Cronbach's alpha value of 0.94.

PA and NA were measured *via* the short form version of the International Positive and Negative Affect Schedule (I-PANAS-SF) (62), a 10-item scale that has demonstrated cross-sample stability, internal reliability, temporal stability, cross-cultural factorial invariance, and convergent and criterion-related validity (62–64). The I-PANAS-SF asks respondents to report on the frequency at which they felt the emotions—such as hostile, upset, inspired, and determined—in the past 2 weeks. Possible responses ranged 1, “never,” to 5, “always.” We summed up the scores of the items that correspond to PA and to NA. Possible PA and NA scores ranged from 5 to 25. In this study, the PA subscale had Cronbach's alpha of 0.76, while the NA subscale had a Cronbach's alpha of 0.87.

Measures for JD-R came from the Questionnaire sur les Ressources et Contraintes Professionnelles (QRCP), a multidimensional scale developed by Lequeurre et al. (31). Given the work of social workers in China, we selected two dimensions of JD (workload and emotional workload) and two dimensions of JR (relationship with colleagues and information) as the measurements for JD and JR in this study. Workload is defined as the sense of having too much work to do in the time available, while emotional workload characterizes emotional job demands, such as needing to cope job-inherent emotions and/or maintaining organizationally desired emotions. Relationship with colleagues refers to team atmosphere and the potential to receive social support from co-workers. Information refers to the availability of information to employees, specifically feedback regarding job performance. Each dimension is measured with 4 items (31). Respondents answered each item on a 7-point Likert scale ranging from 1, “never,” to 7, “always.” Higher scores indicated higher levels of JD or JR. The possible range for each dimension's total score ranged 4 to 28. JD was calculated by summing up the scores of workload and emotional workload, while JR was calculated by calculating the sum of relationships with colleagues and information. In this study, the Cronbach's alpha value of the JD subscale was 0.82. The JR subscale had a Cronbach's alpha of 0.91.

### Analytical Approach

We conducted descriptive and Pearson's correlation analyses to observe the sample characteristics and the correlations among all variables. Then, we conducted structural equation modeling (SEM) analysis to examine the relations among JD-R, PA, and NA, and psychological distress. We selected SEM over regression techniques because it allows for the simultaneous examination of direct and indirect effects through mediating variables (65). STATA software 16.0 was used for all analyses. Results of regression analyses (not shown) using extensive covariates (e.g., personal and family characteristics) indicated that the relations among JD-R, PA, and NA, and psychological distress were similar to those reported here. These results are available upon request.

## Results

Table 2 presents the descriptive statistics of the variables. The sample had an average psychological distress score of 7.2 (SD = 5.2). The average PA and NA scores were 15.7 and 12.1, respectively. The sample reported relatively high JD (M = 38.5, SD = 6.5) and JR (M = 40.8, S.D. = 7.0).

**Table 2:** Descriptive statistics and correlations of key variables.

	**Cronbach's Alpha**	**Mean (S.D.)**	**1**	**2**	**3**	**4**	**5**
1. Psychological distress [0–24]	0.94	7.2 (5.2)	–				
2. Positive affect [5–25]	0.76	15.7 (3.2)	−0.19***	–			
3. Negative affect [5–25]	0.87	12.1 (4.2)	0.44***	0.15***	–		
4. Job demands [8–56]	0.82	38.5 (6.6)	0.15***	0.08;*	0.23***	–	
5. Job resources [18–56]	0.91	40.8 (6.9)	−0.24***	0.31***	−0.21***	0.30***	–

*N = 897. Numbers in parentheses show ranges of the variables*.

* *p < 0.05*,

*** *p < 0.001*.

The Pearson's correlation analysis indicated a positive association between JD and NA (*r* = 0.23, *p* < 0.001) and between JD and psychological distress (*r* = 0.23, *p* < 0.001). Meanwhile, JR was positively associated with PA (r = 0.31, *p* < 0.001) and negatively associated with NA (*r* = −0.21, *p* < 0.001) and psychological distress (*r* = −0.24, *p* < 0.001). PA and NA were significantly correlated with psychological distress (*r* = −0.19, *p* < 0.001; r = 0.44, *p* < 0.001). Surprisingly, JD was positively correlated with PA (*r* = 0.08, *p* < 0.05). Finally, JD and JR were highly positive correlated with each other (*r* = 0.30, *p* < 0.001). Further regression analysis suggests that the positive correlation between JD and PA was driven by JR.

**Figure 2:**
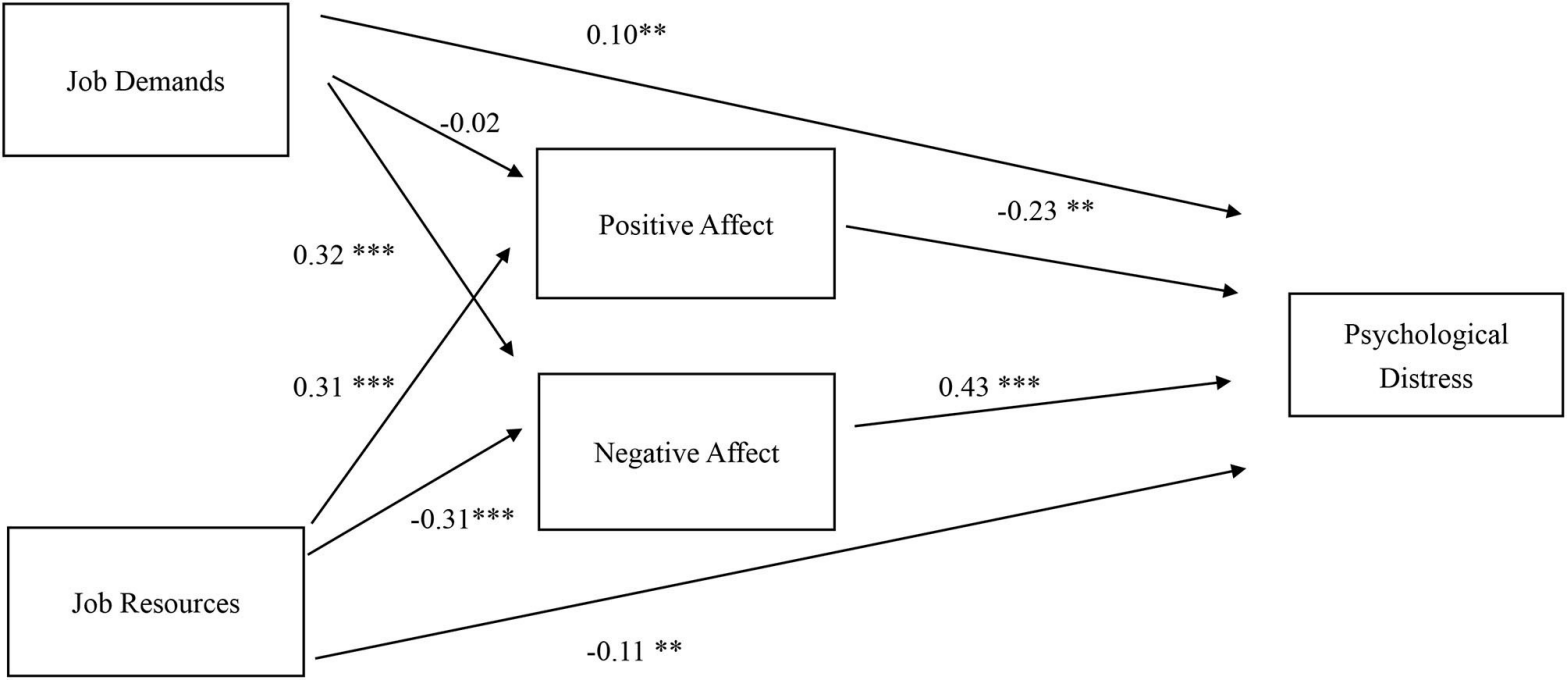
Standardized estimates of JD-R, positive affect and negative affect, and psychological distress. **p < 0.01; ***p < 0.001.

The results of hypothesis testing are presented in Table 3. Figure 2 presents the standardized coefficients of the SEM model. JD had no significant association with PA and was negatively associated with NA (β = −0.32, *p* < 0.001). These results do not support Hypothesis 1 but confirm Hypothesis 2. JR was positively associated with PA (β = 0.31, *p* < 0.001) and negatively associated with NA (β = −0.31, *p* < 0.001). These results support Hypotheses 3 and 4. In addition, both JR (β = −0.11, *p* < 0.01) and PA (β = −0.23, *p* < 0.001) significantly reduced psychological distress, while JD (β = 0.10, *p* < 0.01) and NA (β = 0.43, *p* < 0.001) significantly increased psychological distress. These results support Hypotheses 5 and 6.

**Table 3:** Results of hypothesis testing.

**Hypothesis**	**Beta**	**P**
H1: JD -> PA	−0.02	
H2: JD -> NA	0.32	***
H3: JR -> PA	0.31	***
H4: JR -> NA	−0.31	***
H5: PA -> PD	−0.23	***
H6: NA -> PD	0.43	***
H7: Indirect effect of JD on PD *via* PA and NA	0.14	***
H8: Indirect effect of JR on PD *via* PA and NA	−0.20	***

*N = 897*.

*** *p < 0.001*.

The total effect of JD on psychological distress was 0.25 (p < 0.001) and the indirect effects of JD through PA and NA, together, was 0.14 (p < 0.001). Although JD did not have a direct effect on PA, JD had a significant effect on NA, which in turn had a significant effect on psychological distress. Thus, JD had a significant indirect effect on psychological distress through PA and NA. Proportionately, PA and NA mediated 0.56 (0.14/0.25) of JD's effect on psychological distress. This result provides support for Hypothesis 7. The total effect of JR on psychological distress was −0.32, and the indirect effect of JR on psychological distress through PA and NA (combined) was −0.20 (p < 0.001), or 0.63 of the total effect (−0.20/−0.32). These results support Hypothesis 8.

## Discussion

Given that much of the empirical evidence showing JD-R's effects on work and health outcomes has come from studies focused on Western samples (21, 31, 49, 66), this study extends the literature by investigating JD-R's effects on a sample of Chinese social workers. We applied the JD-R model to examine how JD-R affect psychological distress and whether these relations are mediated by PA and NA. Based on the results of descriptive statistics, the sample of social workers in this study experience high JD in their roles; at the same time, they have considerable JR at their disposal. The SEM results provided support for the hypothesized dual processes by which JD-R affect psychological distress in social workers in China. High JD was associated with high NA and, subsequently, high psychological distress, indicating an energy depletion process (Hypotheses 2 and 6; 21). However, we did not find that JD have effect on PA (Hypothesis 1). Together, the results of JD on PA and NA suggest that JD influence affect through energy depletion process rather than motivational one for this sample. Meanwhile, JR was positively associated with high PA and low NA (Hypotheses 3 and 4), which were both associated with psychological distress (Hypotheses 5 and 6). This indicated a motivational process (21) which protects against psychological distress. The significant indirect effects of JD and JR on psychological distress through PA and NA indicate that PA and NA partially mediated the association between JD-R and psychological distress (Hypotheses 7 and 8). The magnitude of the estimates from our results show that JR have greater effects on PA and NA and psychological distress than JD had on NA and psychological distress. Together, these findings are consistent with and expand upon previous findings with the JD–R model (19–21). Our results show that JD-R are significant predictors of PA and NA and psychological distress, and the underlying energy depletion and motivational processes do not appear to differ between social workers and other professionals.

Based on the findings of our study, we offer a few practice and research suggestions for organizations that employ social workers and researchers who seek to further investigate social workers' occupational well-being or who seek to examine the JD-R model's underlying processes. According to descriptive statistics, the study sample reported high JD. Given that JD significantly and positively predicts NA and psychological distress, employers of social workers in China may seek to focus on both reducing JD and increasing JR to mitigate NA and psychological distress. Descriptive statistics also showed that the sample had considerable JR at their disposal; this shows great promise, given JR's ability to increase PA, reduce NA, and reduce psychological distress. Social work agencies and other employers will need to maintain the availability of JR to their social workers.

Importantly, PA and NA were found to act as mediators of the relations between JD-R and psychological distress, pointing to PA and NA as key points of intervention. To apply this finding, employers may implement a number of interventions that seek to promote PA while reducing NA. For example, studies on mindfulness-based interventions have provided evidence of mindfulness's effectiveness in improving PA and reducing NA (67–69). Moreover, mindfulness-based stress reduction (MBSR), mindfulness-based cognitive therapy (MBCT), and mindfulness-based interventions (MBI) all can effectively reduce psychological distress and promote mental health and well-being (70–72).

While these findings offer a starting point for interventions that bolster the psychological well-being of social workers, they also can act as a guide for further study. Perhaps most notably, our variables of interest—JD-R, PA, and NA, and psychological distress—are all multidimensional constructs that have been measured and operationalized differently throughout the past decades of study. Due to resource constraints, we only used two dimensions to measure JD and JR. While these two dimensions have been found to be significant predictors of workers' outcomes (31), it is necessary that future studies examine the effects of other JD-R dimensions on PA and NA and psychological distress. For example, Demerouti's (21) framework conceptualizes JD with 5 underlying dimensions and JR with 6 underlying dimensions. In Lequeurre et al.'s (31) JD-R framework, JD and JR each consist of 7 dimensions. It is likely that the various dimensions of JD-R and PA and NA will differentially affect each of the psychological distress dimensions, warranting further research.

The results of this study should be considered within the context of several limitations. First, since we collected cross-sectional data from our sample, the results can only approximate associative relations among our variables. To address this limitation, researchers may use a longitudinal design to better examine the causal relations among JD-R, PA, and NA, and psychological distress. Second, our dataset relied on self-reporting from respondents, which leaves the data subject to unintended and intended reporting errors. Social desirability bias, for example, may lead the sample to underreport psychological distress, given the prevalence of stigma related to mental illness in Chinese society (73). Future studies might consider the use of data triangulation, using sources such as colleague reports, employers, and family members. Third, unobserved variables, which were not included in this study, could have influenced on the observed relationships among JD-R, PA, and NA, and psychological distress. For example, we collected data during the global COVID-19 pandemic. Although the COVID-19 cases were under control in China in May 2021, to the extent of ongoing COVID-19 pandemic might have effects on above relationships in Chinese social workers is unknown. Finally, while the sample size and response rate in this study increase our confidence in the results, the generalizability of the findings to all social workers in China is unknown. Our sample was drawn from two districts in Chengdu, one of China's most populous cities [9.3 million in 2021; (74)]. Meanwhile, however, the development and professionalization of social work in China varies by region, due to differences in educational policies and resource allocation (75). Thus, future experimental design should emphasize recruiting a representative sample, as well as examining whether being employed in a rural or urban region may moderate the effects of JDR on PA and NA and psychological distress.

## Conclusion

Applying the JD-R model, this study found that JD and JR affect Chinese social workers' psychological distress through PA and NA. These results add to the growing body of cross-cultural research that supports the dual process by which JD-R affects the well-being of various occupational groups. Social workers have been shown to be a vulnerable occupational group, particularly due to the significant emotional job demands that are characteristic of their work (10, 12–14). We provide evidence of a partial mediation pathway between JD-R and psychological distress through PA and NA, suggesting that PA and NA may be effective points of intervention. The implementation of interventions that reduce NA may serve to buffer against JD's effect on psychological distress as well as to strengthen JR's effect on psychological distress.

## Data Availability Statement

The raw data supporting the conclusions of this article will be made available by the authors, without undue reservation.

## Ethics Statement

The studies involving human participants were reviewed and approved by Review Committee, Research Institute of Social Development, Southwestern University of Finance and Economics. Written informed consent for participation was not required for this study in accordance with the national legislation and the institutional requirements.

## Author Contributions

CH and XX: conceptualization and resources. CH, XX, SC, and YZ: methodology and software, validation, formal analysis, and writing—original draft preparation. CH, XX, and YZ: investigation and data curation. All authors contributed to the article and approved the submitted version.

## Funding

This research and the APC were funded by National Social Science Foundation, China, Grant Number 16BZZ058.

## Conflict of Interest

The authors declare that the research was conducted in the absence of any commercial or financial relationships that could be construed as a potential conflict of interest.

## Publisher's Note

All claims expressed in this article are solely those of the authors and do not necessarily represent those of their affiliated organizations, or those of the publisher, the editors and the reviewers. Any product that may be evaluated in this article, or claim that may be made by its manufacturer, is not guaranteed or endorsed by the publisher.
